# Unsupervised Fault Detection on Unmanned Aerial Vehicles: Encoding and Thresholding Approach †

**DOI:** 10.3390/s21062208

**Published:** 2021-03-22

**Authors:** Kyung Ho Park, Eunji Park, Huy Kang Kim

**Affiliations:** Graduate School of Cybersecurity, Korea University, Seoul 02841, Korea; kyungho96@korea.ac.kr (K.H.P.); epark911@korea.ac.kr (E.P.)

**Keywords:** Unmanned Aerial Vehicle, fault detection, anomaly detection, unsupervised learning, autoencoder

## Abstract

Unmanned Aerial Vehicles are expected to create enormous benefits to society, but there are safety concerns in recognizing faults at the vehicle’s control component. Prior studies proposed various fault detection approaches leveraging heuristics-based rules and supervised learning-based models, but there were several drawbacks. The rule-based approaches required an engineer to update the rules on every type of fault, and the supervised learning-based approaches necessitated the acquisition of a finely-labeled training dataset. Moreover, both prior approaches commonly include a limit that the detection model can identify the trained type of faults only, but fail to recognize the unseen type of faults. In pursuit of resolving the aforementioned drawbacks, we proposed a fault detection model utilizing a stacked autoencoder that lies under unsupervised learning. The autoencoder was trained with data from safe UAV states, and its reconstruction loss was examined to distinguish the safe states and faulty states. The key contributions of our study are, as follows. First, we presented a series of analyses to extract essential features from raw UAV flight logs. Second, we designed a fault detection model consisting of the stacked autoencoder and the classifier. Lastly, we validated our approach’s fault detection performance with two datasets consisting of different types of UAV faults.

## 1. Introduction

The Unmanned Aerial Vehicle (UAV) has developed in recent years, pursuing a wide range of applications, such as traffic surveillance [1], delivery [2], or environment exploration [3]. The UAVs perceive the flying environment with embedded sensors, receive commands from the base station or autonomous flight algorithm, and fly toward the destination by providing inputs to control the vehicle’s components. However, UAVs are evaluated as yet to be actively utilized in society due to safety and reliability concerns. One of the safety concerns is regarding cybersecurity [4]. UAVs go out of control if the communication signals are intruded by malicious attackers, and the UAV would fail to stabilize its body in the air. Not only the fault caused by cyberattacks, the fault on physical components such as the aileron also makes the UAV go out of control. As the occurrence of a fault on UAVs creates a tremendous amount of damage, a fault detection model is definitely required for the sake of safety and reliability [5,6]. Following the aforementioned safety concerns, the industry and academia have studied a fault detection model to identify the occurrence of a fault on the UAV.

The early studies on UAV fault detection are rule-based approaches [7,8,9]. Early researches scrutinized that fault patterns are logged in a system log of the UAV and established fault detection rules. Because the engineer can set the fault detection model by setting a fault detection rule on the system, these rule-based approaches were advantageous in implementation. However, rule-based approaches had several limits to be deployed in the real-world, as described in [10]. First, rule-based approaches’ fault detection performance was not precise enough as the model fails to identify fault which was not modeled by rules. Second, the engineer should update detection rules for each type of system failure, and it created a particular resource consumption on model maintenance. In pursuit of resolving the aforementioned drawbacks of rule-based fault detection approaches, academia and industry sought improved approaches with better detection performance and less maintenance effort.

A fault detection method that is based on analytical models using signatures can be a part of that research. The approach allows the detection model to predefine features that are associated with the signature for known patterns of cyberattacks. The signature-based detection method is far more advantageous for maintenance than the rule-based approach, which requires specific rules for all behaviors one-by-one [5,11]. Shoufan et al. [12] devised authentication for UAV flight command data, called Behaviometric. The study considered the combinations of the flight command set and the UAV’s behaviors as signatures, and then used them to detect malicious commands from the attackers. However, this technique has the disadvantage of only being applicable for known fault patterns. When considering the limitations of diverse approaches, the learning-based method of detection models is a big resonant with research.

Rcent progress in machine learning and neural networks has opened a novel approach to UAV fault detection [13,14,15]. As machine learning and deep neural network models can effectively learn the distinctive pattern of fault with unexpected inputs on the UAV [16], past researches cast the fault detection task as a binary classification problem between fault and safe status. The proposed studies acquired a flight dataset, which is finely labeled along with the type of faults. They trained the detection model in a supervised manner and achieved precise fault detection performance. These supervised learning-based approaches resolves the drawbacks of rule-based approaches and signature-based approaches by effectively identifying the faults on the UAV, including unknown security threats [17]. The learning-based approaches did not necessitate rules to every type of faults and accomplished a precise detection performance.

Although the supervised learning-based methods achieved a precise fault detection performance, there existed drawbacks for real-world application. Zhou et al. [18] introduced weakly supervised learning due to the following drawbacks. First, supervised learning-based methods require heavy effort in finely-labeled dataset acquisition. Because learning-based fault detection models are trained in a supervised manner, the model requires an engineer to provide the data and labels as a pair. The engineer should collect log data, which include faults during the flight, but we analyzed whether this dataset acquisition step requires a resource consumption. Second, the supervised learning-based models cannot identify faults if the fault is not trained during the model training step. Because the trained model only learned the pattern of faults included in the training dataset, the model might fail to recognize a novel type of faults.

This study proposes a fault detection model utilizing an autoencoder, one of the representative unsupervised learning models. First, we only trained the autoencoder with flight data under safe states to let the model learn safe UAV states’ patterns. Note that the training stage employs an unsupervised learning paradigm, as it requires a single class of data: safe UAV states. The trained model was scrutinized to produce the distinctive reconstruction loss between safe states and faulty states. The well-trained model was expected to produce low reconstruction loss with safe state data. On the other hand, we expected the trained model to generate a large reconstruction loss with faulty states’ data because the faulty states’ pattern is not learned. We figured out that this distinctive level of reconstruction loss between safe states and faulty states can be a significant cue of fault detection and examined our approach through a series of experiments.

As an expansion of the study proposed in [19], the contributions of our study are as follows:

- When considering the flight as a part of a cyber physical system (CPS), many concerns that are related to the cybersecurity on the system have been raised, according to Sanchez et al. in [20]. We proposed a series of analyses to extract an essential set of features from raw log-level flight data and validated that the set of features represents a distinct pattern between safe states and faulty states. The details are shown in Section 3.3.
- We established a fault detection model leveraging an autoencoder by only training the safe state data. The proposed design of the fault detection model, which is described in Section 3.4, let the engineer evade an effort of finely-labeled dataset acquisition. Moreover, the model can identify unseen types of faults, despite the pattern of faulty states not being trained.
- We examined the detection performance of the proposed approach with two types of UAV fault datasets, as specified in Section 3.1. One dataset consists of flight logs under the fault occurrence that is derived by cyberattacks, and the other dataset includes flight logs under the fault from the failure of physical components. Throughout the experiments, the proposed fault detection approach validated the capability of identifying faults that are caused by cyberattacks with no recovery measures and physical component failure with recovery measures. The experimental results are shown in Section 4.3 and Section 4.4.

## 2. Literature Review

In this section, we reviewed prior studies on fault detection approaches for the UAV, where faults occurred at the system’s control components. Our study set the fault detection model’s scope that identifies failures on the control component, not the vehicle’s physical components. Following the type of used algorithm in the study, previous works were categorized into three categories: rule-based approaches, supervised learning approaches, and unsupervised learning approaches.

### 2.1. Rule-Based UAV Fault Detection

Early studies of fault detection employed heuristics-based rules to capture distinct characteristics of faulty states from safe states. Note that the early researches focused on fault detection that was caused by intrusions from malicious attackers. Mitchell and Chen [7] led a pioneering study of developing behavior UAV rule-based intrusion detection systems (BRUIDS). Sedjelmachi et al. [8] conducted studies to detect faults that were caused by attacks on the ad-hoc network of UAVs. They proposed a lightweight Intrusion Detection System (IDS) that detects integrity attacks and Denial of Service (DoS) attacks fast in such situations. The IDS minimized the overhead of the communication network and showed precise fault detection rates. Muniraj et al. [9] proposed a framework to recognize faults from cyber-physical attacks targeting UAVs. They investigated the UAV’s sensors to determine which sensors were vulnerable to specific attacks and established the particular diagnosis rules on the detection system. The simulated experiments were conducted with GPS spoofing attacks, and the detection process classified an input state of the UAV as faults when sensors responded to the request in a distinctive manner. In the context of early studies on UAV fault detection, rule-based approaches achieved promising detection performance and provided a concrete motivation to the following studies.

### 2.2. Fault Detection with Supervised Learning

Previously-illustrated fault detection approaches identified faults that were caused by cyberattacks through establishing particular detection rules on UAV behaviors or sensor states. However, these approaches contained a drawback that rule-based approaches could not capture the unknown type of faults, and it required engineers to establish new rulesets for the new type of faults or attacks. In pursuit of improving this drawback, data-driven fault detection systems were proposed. The data-driven fault detection approaches have utilized a supervised learning paradigm that leans on UAV states’ pattern from the finely-labeled dataset.

Bronz et al. [13] proposed a system that detects faults in real-time in the in-flight state of UAV. The detection system utilized Support Vector Machine (SVM), which is one of the machine learning algorithms known to be effective on classification problems. The suggested fault detection approach had the advantage of defending the false alarm by reflecting the possibility of geometric errors in small UAVs in the detection. Arthur [14] studied a fault detection method to identify anomalies from signal spoofing and jamming attacks on lightweight UAVs. UAVs perform a self-analysis and self-detection under the proposed fault detection scheme while using the multi-class SVMs with significant features that were identified through the self-taught learning (STL) algorithm. Kim et al. [15] used machine learning techniques to detect faults from cyberattacks, such as sensor spoofing. They identified the issue that learning-based detection models require a large amount of data and attempted to increase data using a generative adversarial network (GAN).

### 2.3. Fault Detection with Unsupervised Learning

While the supervised learning-based approaches achieved significant fault detection performance, the supervised paradigm requires well-labeled data. Furthermore, the supervised learning-based detection approaches cannot identify the unseen type of faults, as the model did not learn their pattern. To resolve these drawbacks, recent researches with learning-based detection systems utilized unsupervised learning with unlab5678eled data. The fault detection system solves a binary classification problem between the safe state and faulty state, leveraging unsupervised learning. A primary paradigm of unsupervised fault detection cast the problem into anomaly detection to identify safe states only and then classify any other abnormal patterns as faulty states.

Xia et al. [21] presented an UAV faults detection model employing a semi-supervised paradigm. First, they established a stacked denoising autoencoder (SDA) and trained the model with unlabeled data to learn the flight data patterns. Second, they trained a classifier in a supervised manner with a few labeled dataset. Throughout experimental analyses, they showed the use of a trained autoencoder as a feature extractor contributed to the fault detection performance. Still, the proposed approach highly relied on a supervised manner. Whelan et al. [22] defined fault detection task as a novelty-based detection. They conducted research using various one-class classifiers, including One-class SVM (OC-SVM), Autoencoder, and Local Outlier Factor (LOF). Note that the LOF was the only unsupervised learning-based algorithm. The motivation behind their use of the classifier was that the supervised learning-based approaches might not be efficient enough in situations where the fault’s landscape depends on activated payloads or sensors change and labeled data acquisition encounters challenges. The LOF was a density-based method for detecting the novelty on the unlabeled input data. They experimented with fault cases demonstrated precise detection performance. Khan et al. [23] pursued unsupervised learning, and their UAV fault detection system was based on the distance between state vectors at safe states and faulty states. They intended to detect attacks without requiring much prior knowledge of how changes in data by attacks. The learning models that were used in the research were one-class SVM and distance-based models, including the Gaussian mixture and Mahalanobis distance models. They exploited time-series sensor data to catch abnormal data from the clustered distributions.

## 3. Proposed Methodology

**Figure 1:**
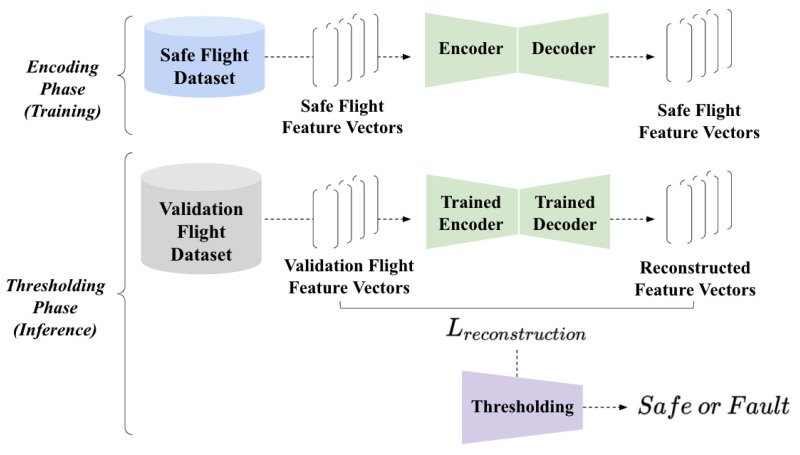
The overall structure of the proposed fault detection model. During the encoding phase, we acquired safe flight logs and extracted feature vectors from the logs, and trained the autoencoder to scrutinize safe flight patterns. During the thresholding phase, our approach takes a validation feature vector that is acquired from the validation flight logs, provides the input to the autoencoder, and acquires the reconstruction loss. If the reconstruction loss is larger than the particular threshold, then our approach identifies the Unmanned Aerial Vehicle’s (UAV’s) fault.

This section illustrates a series of analyses illustrating how we designed the fault detection model with unsupervised learning. Following the literature review illustrated in a prior section, our study is primarily motivated by unsupervised learning only approaches. The fault detection model contained two parts: the encoding phase with model training and the thresholding phase that classifies an input between the safe state and the faulty state. Figure 1 visualizes the proposed fault detection model’s overall structure. Starting from the dataset acquisition, we elaborate how an essential set of features are established, and the autoencoder is utilized to identify faultystates from safe states.

### 3.1. Dataset Acquisition

#### 3.1.1. Description

Throughout the study, we analyzed the consideration of various faulty circumstances would contribute to the development of a robust UAV fault detection model. In order to examine our approach’s effectiveness in various faulty situations, we categorized two types of fault from the prior studies described in Section 2: fault without recovery measures and the fault with recovery measures. Following these fault types, we utilized two types of UAV fault detection datasets: the UAV Attack (UA) dataset and AirLab Failure and Anomaly (ALFA) dataset. We illustrated the description of datasets below.

**UA dataset: Fault from Cyberattacks without any recovery measures.** The UA dataset consists of the quadcopter’s raw flight logs and the fault occurred from cyberattacks to the UAV system. The UA dataset contains a quadcopter flying in a simulated environment. The dataset simulates a flight scenario that the quadcopters flew with control inputs from the base station’s Autopilot commands, and the related control components, such as the elevator or engines activate following the provided command. The dataset includes system logs along with a flight on the simulator, which follows a conventional jMAVSim setup. The UA dataset contains three types of flight logs: safe flight, flight under DoS attack, and flight under GPS Spoofing attack. We analyzed that the UA dataset describes severe faults on the system as the UAV got down and crashed to the ground without any recovery measures after the cyberattack. This study employed the UA dataset to identify the fault on the UAV when the recovery measure is not activated after the fault happened. Note that the UA dataset includes annotations on UAV states between the safe state and the faulty state. We utilized flight logs under the safe state during the encoding phase, which trains the model under the unsupervised learning paradigm. During the thresholding phase, which performs an inference, we utilized flight logs from both safe states and faulty states to measure the detection performance. The detailed description of the UA dataset is illustrated in [22].

**ALFA dataset: Fault from Control Component Failure with recovery measures.** The ALFA dataset is a fixed-wing UAV’s raw flight logs, and control component failures cause the fault. The failures on the fixed-wing UAV’s control components are the incapacity in rudder, aileron, elevator, and engine. While the UA dataset contains flight logs in a simulated environment, the ALFA dataset provides a flight log in real test flights with actual control component failure. Note that experimental real-world flights are performed at the airport in Pittsburgh, the United States. The ALFA dataset provides five types of flight logs: safe flight, a flight under failures at the aileron, rudder, engine, and the elevator. Note that the human safety pilot provided adequate recovery measures to the UAV after the physical component failure happened. Throughout the study, we utilized the ALFA dataset to recognize faults when the UAV still flies, but included a fault on the system body. Note that [24] describes the detailed description of the ALFA dataset.

#### 3.1.2. Ground Truth Confirmation

The ground-truth confirmation is the first and foremost step before a fault detection model establishment. We labeled every log in safe flights in both the UA dataset and ALFA dataset as ‘safe’ because there exists no failure during the flight. For the faulty flight data, both the UA dataset and ALFA dataset provided a timestamp when the fault happened to the UAV so that the labels on the logs before the fault happened became ‘safe’ and logs after the fault happened labeled as ‘fault’. Note that the sanity of ground truth on the dataset was clarified and assured before implementing the fault detection model.

### 3.2. Feature Selection

The raw flight logs recorded in UAVs include a wide range of features. We categorized various features into five categories: Location, Position & Orientation, Internal Measurements, System Status, and Control. The descriptions of each category are described below.

- **Location:** a set of features related to the location of the UAV. A particular coordinates of the location is described along with the GPS
- **Position & Orientation:** a set of features related to the position and the orientation of the UAV.
- **Internal Measurements:** a set of features extracted from the Internal Measurement Units (IMUs).
- **System Status:** a set of features related to the system management, such as on-board sensors.
- **Control:** a set of features illustrating an input toward the actuator

Our data-driven fault detection approach’s key assumption is that features that are measured from each UAV component presumably include distinct patterns of faulty states distinct from safe states. Our approach aims to learn safe UAV states’ characteristics from these features during the encoding phase and recognize any abnormal patterns as faults during the thresholding phase. While these raw flight logs include patterns of UAV states, several missed features or non-essential features exist for fault detection. To evade a curse of dimensionality and extract essential information for fault detection, the feature selection process was crucial in designing an input feature vector. Based on the aforementioned analogy, we established two rules for the feature selection step: hardware generality and sensor stability. From the raw features of the dataset, the selected features were those not filtered by both rules.

**Rule 1: Hardware Generality.** The rule of hardware generality aims to eliminate the features that are unique in particular UAV hardware. We evaluated that a fault detection model should be easily implemented regardless of the hardware. Suppose a feature that only exists at a particular UAV is employed in our detection model. In that case, the model cannot be applied to other UAV types as the feature does not exist. Therefore, every unique feature that only exists at a particular type of UAV was excluded. As a representative case, the feature related to an actuator control was not selected, as the control input varies with the UAV type. For instance, a control input to the actuator differs at the quadcopter and the fixed-wing UAV; thus, the related features harm the hardware generality. Under the consideration of this rule of hardware generality, we excluded feature in the category of **Location** and **Control** on this study and focused on the physical properties of the UAV during the flight.

**Rule 2: Sensor Stability.** The second rule for the feature selection is sensor stability. Suppose the case where a particular feature is frequently not recorded during the flight. The feature vector’s lost value lets the feature vector include a NULL value, which becomes an obstacle to the model training and fault detection during the flight. Furthermore, suppose another case where a particular feature does not change at all during the flight. The tranquil features do not provide any meaningful information to detect faults on the UAV, but rather increase the curse of dimensionality; thus, we decided to eliminate this tranquil feature. Throughout the consideration of sensor stability, every feature that satisfies the following conditions concluded not being included.

- **Condition 1:** the feature contains any Null value during the flight.
- **Condition 2:** the feature does not change at all during the flight.

We applied the aforementioned feature selection rules to every feature at raw flight logs in both the UA dataset and ALFA dataset. We resulted in the list of selected features in Table 1 and Table 2 for the UA dataset and the ALFA dataset, respectively. Note that the number of features at the UA dataset is larger than the ALFA dataset, as the UAV dataset generated enormous raw flight logs on the simulator while the AlFA dataset recorded the flights in the real world.

### 3.3. Feature Engineering

We employed feature engineering processes to transform flight logs into the feature vector after we selected the essential features. The feature engineering includes two sub-steps: the feature scaling and timestamp pooling.

#### 3.3.1. Feature Scaling

The feature scaling step is designed to unify the range of values at each feature. Because individual features are recorded in a different magnitude of the scale, we expected that a fault detection model consisting of deep neural networks would become confused easily during the parameter optimization. The Min-Max scaling function elaborated that is in Equation (1) was utilized in the study to scale each feature under the same scope between 0 and 1.

(1) $$X_{scaled}=\frac{X_{i}-Min\left(X_{i}\right)}{Max\left(X_{i}\right)-Min\left(X_{i}\right)}$$

#### 3.3.2. Timestamp Pooling

**Figure 2:**
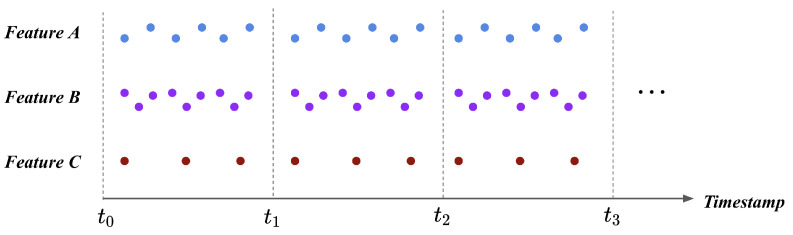
Flight logs before timestamp pooling. Along with the feature type, there exists a different number of values during a single time window. As the model receives a fixed-size input, timestamp pooling is required to transform the flight logs into the fixed-size feature vector.

After each feature’s scale became unified, the scaled flight logs necessitate the process of transformation into the fixed size. When we consider input to the fault detection model, the input should be a feature vector in a fixed size that describes the UAV’s status at the particular timestamp. However, individual features are recorded in the UAV in different periods; thus, flight logs cannot be directly transformed into the feature vector as an input. Figure 2 illustrates the problem. Referring to Figure 2, the feature A, B, and C are recorded in different periods to the UAV at the same time window. Suppose the case where we transform these features during a particular time window. As the length of features varies during the same time window, the number of data points at each feature is 6, 9, 3 for the feature A, B, and C, respectively. The number of feature values during a single time window should be the same, but the current flight logs cannot earn this requirement.

**Figure 3:**
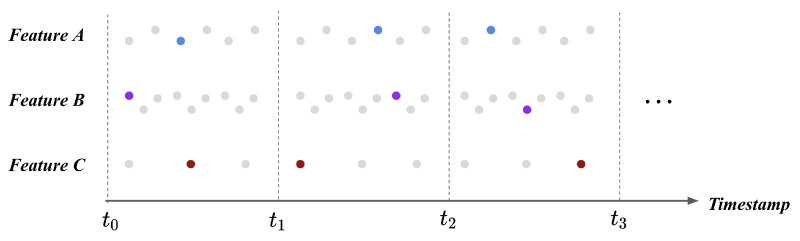
Flight logs after timestamp pooling. We selected a single representative value during the particular time window to make each feature includes the same number of value. The timestamp pooling transforms flight logs into the fixed-shape feature vector during a single time window; thus, the produced feature vectors can be provided to the model.

We utilized timestamp pooling to resolve the different length of each feature during a single time window. The timestamp pooling method randomly selects a single data point during a fixed time window. We extracted a random feature value and assumed that the selected value could represent the feature during the time window. The time window was set as 250 milliseconds, and timestamp pooling was applied to every feature. Figure 3 illustrates the result of timestamp pooling at the example case. Each feature is represented with a single value during a time window; therefore, flight logs transform into the feature vector in a fixed size. The feature vectors lie under the same scale and same shape; thus, it resulted in the processed feature vector being provided as an input to the fault detection model.

### 3.4. Fault Detection Model

#### 3.4.1. Autoencoder

Based on the processed feature vector, we established a fault detection model leveraging the autoencoder. The autoencoder is a deep neural network that encodes the input feature vector and decodes to reconstruct the given feature vector. The autoencoder’s encoding part optimizes its parameters to produce a representation vector, including the feature vector’s essential characteristic. The decoding part optimizes its parameters to reconstruct the given input feature vector from the produced representation vector. A loss function is set as the difference between the original input vector and reconstructed vector; thus, minimizing the loss allows the autoencoder learn to compress the input feature vector as a representation vector [25]. This study utilized a stacked autoencoder, where multiple layers of neurons are stacked at both the encoder and decoder. A single layer of neuron computes a linear operation to the input vector and applies the activation function of the ReLU to add non-linearity to the model [26].

For the sake of clarified elaboration on the model, we mathematically described a single layer of the encoder, a single layer of the decoder, a loss function (reconstruction loss), and the objective function at Equations (2)–(5), respectively. For the terms that are used in the equations above, e() and d() imply the encoder and the decoder, respectively. *x* denotes the input feature vector that is provided to the encoder, *r* denotes the representation vector provided to the decoder. *W* and *b* denote the weights and biases of the linear operation, and ReLU implies a non-linear activation function proposed in [26]. θ denotes the overall parameters of the encoder and decoder, and $f_{\theta }$ denotes the overall model consisting of the encoder and the decoder. *y* implies the ground-truth vector for the model, which is equal to the given input feature vector (*x*), following the unsupervised learning paradigm.

When considering Equations (2) and (3), a single layer of the encoder and the decoder takes an input vector, performs a linear operation with weights (*W*) and biases (*b*), and results in an output vector after applying the ReLU function as an activation. Because the ReLU is a non-linear activation function, we expect that it empowers the model to scrutinize patterns that exist at the feature vector effectively [26]. Equation (4) implies a mean squared error between an input vector and the reconstructed vector. Note that *n* denotes the number of layers at the encoder and decoder, and the model consists of stacked encoder layers and the decoder layers under the same number. Equation (5) illustrates the model training objective that aims to let the stacked autoencoder finely understand the underlying feature dynamics of the UAV states and reconstruct the given input without much loss. We set the optimal parameters of the model as $\theta^{*}$, which minimizes the total loss at the given *x* from the dataset *D*. Please check all letters and keep them with same expressions. such as italic or normal.

(2) $${\mathrm{Encoder}:e(x)=\mathrm{ReLU}(W}_{encoder}x+b_{encoder})$$

(3) $${\mathrm{Decoder}:d(r)=\mathrm{ReLU}(W}_{decoder}r+b_{decoder})$$

(4) $$\begin{array}{c} {\mathrm{Loss}:L(x,y)=||f}_{\theta }\left(x\right)-y{\left|\right|}^{2}\;where\;f_{\theta }\left(x\right)=d^{n}\left(e^{n}\left(x\right)\right) \end{array}$$

(5) $$\mathrm{Find}\;\theta^{*}\;s.t.\;\;\theta^{*}=argmin_{\theta }\left(\sum_{x\in D}L\left(x,y\right)\right)$$

#### 3.4.2. Encoding Phase: Learning the Pattern of Safe States

Our fault detection model’s key motivation is as follows: the stacked autoencoder trained only with safe flights would produce a different level of reconstruction losses between safe states and faulty states. Because the training phase is provided the feature vectors from the safe flights, the autoencoder naturally learns safe flights’ patterns only. The parameters of the autoencoder are optimized to encode and decode the safe states, but not optimized to produce a proper representation of faulty states. In other words, the trained autoencoder yields low reconstruction loss when we provide a feature vector of safe states but results in larger reconstruction loss on the feature vector of faulty states. We analyzed that this disparity on the reconstruction loss between safe states and faulty states can be a useful cue of fault detection. Therefore, the autoencoder was trained with feature vectors from safe flights only to achieve the aforementioned cue of the fault detection. Note that we did not use any feature vectors from the faulty flight log, but we only utilized feature vectors from safe flight logs during the encoding phase, which trains the model.

#### 3.4.3. Thresholding Phase: Fault Detection

Last but not least, we identified faulty states during the validation phase by setting a particular threshold level. By setting a particular level of threshold, our inference was that a given input feature vector was a fault if the reconstruction loss of the vector becomes larger than the threshold. On the other hand, we classified a given input feature vector as a safe state when the vector’s reconstruction loss stays lower than the threshold. Assuming that the autoencoder is properly trained, this simple approach of thresholding the reconstruction loss can effectively identify faulty states during the inference phase. Note that the fault detection performance might vary along with different threshold levels; therefore, the fault detection method successfully resolved the aforementioned problem, as illustrated in the following section.

## 4. Experiments

### 4.1. Experiment Takeaways

We clarified experimental takeaways that describe research questions to examine with a series of experiments. The key experiment takeaways of our study are illustrated below. While our previously-proposed study [19] employed a single dataset, note that we extended the prior work by employing another dataset where recovery measures are followed after the fault happened.

- **Fault Detection on the Flight without Recovery Measure.**
  Suppose that the case when the UAV cannot provide an adequate recovery measure after the fault happened. The UAV system goes out of control and it might fail to balance its body in the air. In this case, a fault detection model should identify the occurrence of a fault on the UAV and notify the proper supervisor. The UA dataset describes a fault without automatic recovery measures that are caused by cyberattacks; thus, we employed the UA dataset in this experiment takeaway to examine whether the proposed model can identify faults from safe states.
- **Fault Detection on the Flight with Recovery Measure.**
  Suppose that the case when the automatic recovery module provided adequate recovery measures to the UAV body when the physical component failure happened. As UAVs’ crash creates enormous damage, modern UAVs have the ability of self-recovery to use an alternative physical component or land on the ground safely when the physical component failure happened. In this case, a fault detection model shall identify the fault and report to the supervisor, even if the UAV still maintains the flight in the air. Because the ALFA dataset illustrates a fault with automatic recovery measures caused by a physical sensor failure, we utilized the ALFA dataset in this experiment takeaway to validate whether our approach recognizes faults.

### 4.2. Evaluation

As we set a particular threshold during the inference phase, our model’s detection performance would vary along with the level of threshold. The evaluation metrics should resolve this problem to evaluate the model performance, regardless of the threshold level; thus, the Area Under the Curve (AUC) at the Receiver Operating Curve (ROC) is chosen as a key evaluation metric. The ROC curve is a probability curve that takes a false positive rate on the *x* axis and a true positive rate on the *y* axis. It graphically shows a different level of detection performance along with threshold levels. The AUC implies the area under the ROC curve, and it represents a classification performance on various threshold levels in a numeric measurement. The higher AUC measurement represents the model solving the classification task better. Because the AUC numerically describes the classification performance, we employed the AUC as a key evaluation metric to illustrate the fault detection performance for various threshold levels.

### 4.3. Fault Detection on the Flight without Recovery Measures

We examined the first experiment takeaway, which asks the fault detection performance at the flight with faults under no recovery measures. First, the training set consisted of feature vectors that are extracted from the safe flight only. We trained the autoencoder with the aforementioned training set to let the model learn the pattern of safe UAV states. Second, two test sets took feature vectors that were derived from the flight with the fault from GPS spoofing attack and DoS attack, respectively. The flight log under the fault contains both safe states and faulty states; thus, the fault detection model solved a binary classification task between the safe state and fault. We provided this test set to the trained autoencoder and acquired the reconstruction loss. Finally, the fault detection performance was evaluated along with various threshold levels with the AUC level. Note that Table 3 describes the test set’s configuration and the fault detection performance on each test case.

**Figure 4:**
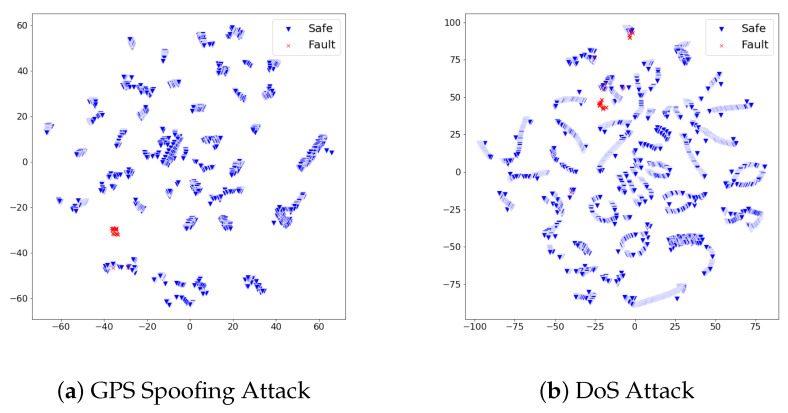
The distribution of dimensionality-reduced feature vectors from the safe states and faulty states vary on the two-dimensional (2D) space. The different distribution of both states implies the set of used features effectively describes each state’s pattern. We interpret this distinct distribution between safe states and faulty states empowered the autoencoder to effectively focus on patterns of safe UAV states; therefore, our approach could achieve precise fault detection performances in the UA dataset.

We analyzed whether the proposed fault detection approach precisely identified faults of GPS spoofing and DoS attacks from safe states. In pursuit of discovering the underlying reason for the performance, we reduced the high-dimensional feature vectors of both safe and faulty states into the 2D space with t-sne [27], as visualized in Figure 4. The distribution of feature vectors of faulty states formed a cluster that differs from safe states. We interpret the proposed set of features on the UA dataset to effectively describe the unique pattern of safe states and faulty states. The autoencoder would not confuse safe feature vectors from the faulty feature vectors due to their distinct distribution; thus, it empowered a precise fault detection performance. Throughout the experimental results on the UA dataset, we examined the experimental takeaway that our fault detection model can recognize faults in the flight where the recovery measure is not provided to the UAV after the fault happened.

### 4.4. Fault Detection on the Flight with Recovery Measures

We also examined whether the proposed fault detection model precisely recognizes faulty states when the automatic recovery measures are applied after the fault happened. The training set consisted of feature vectors that are extracted from the safe flight and trained the autoencoder with the aforementioned training set. The test set was configured to be composed of four types of fault from physical component failures: rudder failure, elevator failure, aileron failure, and engine failure. Note that each test set includes both safe states and faulty states. We provided these four test sets to the trained autoencoder and extracted the reconstruction losses. Following the evaluation metric, the AUCs of the fault detection results were calculated and are denoted in Table 4.

**Figure 5:**
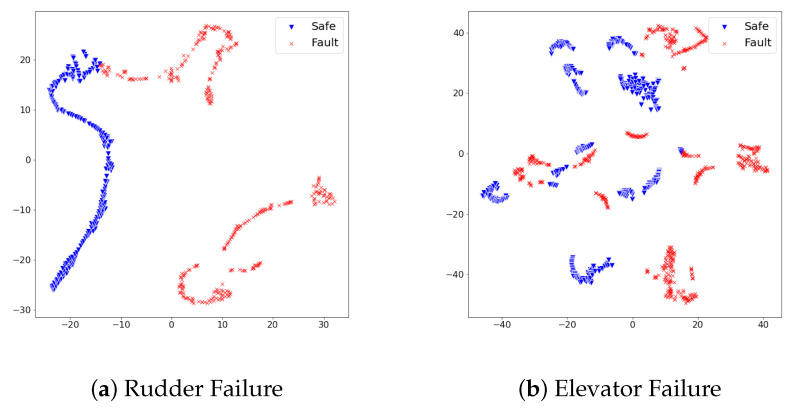
The distribution of dimensionality-reduced feature vectors from the safe states and faulty states vary on the 2D space. The different distribution of both states implies the set of used features effectively describes each state’s pattern. We analyzed how the lesser adjoined distribution between safe states and faulty states contributed to precise fault detection performance.

**Figure 6:**
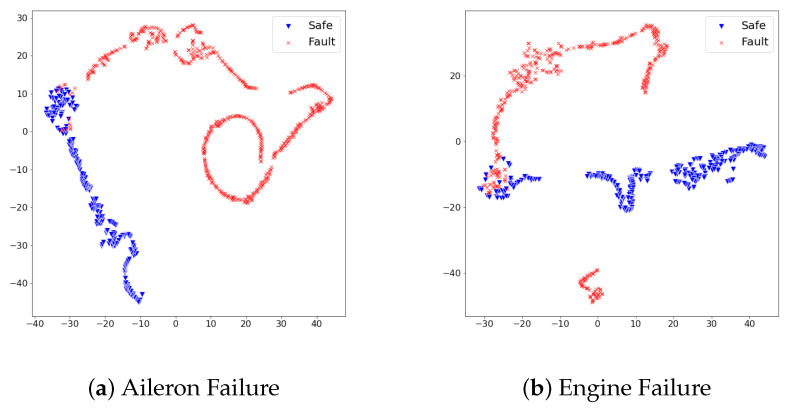
The distribution of dimensionality-reduced feature vectors from the safe states and faulty states vary on the 2D space. The duplicated area consists of feature vectors from safe states and faulty states imply that the fault detection model would confuse the identification of faulty states from safe states. We analyzed this adjoined distribution between two states hampered our approach to accomplish a promising detection performance.

When referring to Table 4, the fault detection model achieved promising performance on test sets of rudder failure and elevator failure. On the other hand, the detection performance on test sets of aileron failure and engine failure required an improvement to be deployed in the real world. We scrutinized the reason for different detection results from the distribution of feature vectors in test sets. In the same dimensionality reduction approach with t-sne, the feature vectors of each test set turned into 2D space and visualized them. Figure 5 and Figure 6 show the visualized results.

In Figure 5, feature vectors of safe states and faulty states distribute distinctly; thus, we analyzed the proposed detection model produced large reconstruction losses on faulty states, while it produced lower reconstruction losses on safe states. On the other hand, Figure 6 illustrates the distribution of safe states and faulty states at aileron failure and engine failure was not as effective as rudder failure and elevator failure. Although safe states and faulty states are divided, there commonly exists a duplicated area between two states. This duplicated area implies that particular feature vectors look similar in the model’s point of view; thus, the model would confuse faulty states and safe states under the given set of features. Along with the analysis on feature distribution of four test sets, we found that the proposed set of features at the ALFA dataset can contribute to the fault detection model in a particular way; however, there is a room for improvement in achieving better detection performance.

### 4.5. Analogy

Throughout the series of analyses with two experiment takeaways, examining the fault detection task is more challenging when the recovery measure is applied after the fault happened. The fault detection task can be comparatively easy when the recovery measure is not provided after the fault occurs. The faults derived from cyberattacks let the UAV go out of control without adequate recoveries; thus, the UAV would fail to sustain its flight and shudder in the air or even start to fall down to the ground. We estimates this severe damage caused by the absence of recovery measures highlighted the pattern of faulty states and contributed to precise detection performance.

On the other hand, analyzing the fault detection task becomes harder when the recovery measure is applied to the UAV. As the recovery measures let the UAV sustain its flight in the air, our inference headed to the fact that the set of proposed features were not perfect enough to identify faults from the UAV that holds recovery measures. The recovery measures at least prevent the UAV from a severe problem, such as a crash to the ground. However, there still exists a necessity for fault detection to assure safety and reliability to understand the UAV’s state as correctly as possible. In a nutshell, our fault detection approach resulted in effectively identifying the faulty states when the UAV is not prepared with recovery measures, but it requires room for improvement when the recovery measure is embedded into the vehicle body.

## 5. Discussions

Throughout the study, we examined the proposed approach achieved a promising performance on fault detection tasks. Nevertheless, our approach requires further contemplation on deploying the detection model into real-world UAVs. The detailed elaborations of room for improvement are as follows.

### 5.1. Improving the Fault Detection Performance

The proposed approach necessitates the extended study of improving detection performances. First, we expect a further extension of features to elevate the detection performance. Along with the series of analyses, we discovered that the proposed set of features could describe the distinct pattern of safe states and faulty states. While the study employed the UAV’s physical properties as features, further study might utilize network properties that are recorded during the communication between the UAV and base station for the fault detection task. We especially discovered that fault occurrences with recovery measures are more challenging for the detection model with features describing the physical properties of the UAV. The exploratory analyses on the change of network properties would provide a new set of features for performance elevation.

Second, we infer that further variations of the autoencoder would contribute to better fault detection performance. The feature dynamics in a single timestamp were meant to illustrate the UAV’s state. The focus of the dynamics might move to other types of features, such as the sequential features of the flight. Because the flight logs are recorded sequentially, the expected pattern is also sequential, which effectively demonstrates a difference between safe states and faulty states. We expect that the input feature vector can be transformed in a sequential manner, and the stacked autoencoder can be changed into the sequential autoencoder leveraging recurrent neural networks [28]. Under the paradigm of utilizing unsupervised learning, we expect that further studies can design the fault detection model to scrutinize distinct patterns between safe states and faulty states on the UAV.

### 5.2. Computation Overhead on Real-World UAVs

We expect that the proposed approach shall be examined on real-world UAVs to measure the computation overhead during the inference phase. The fault detection model would be implemented in the UAV and classify the state between the safe states and faulty states. A computing device with Graphical Computing Units (GPUs)—which is employed in our study—was capable enough to perform binary classification on the test set. However, the proposed detection model might create a particular burden of computation overhead on real-world UAVs’ computing environment. We analyzed whether the following studies on our research would be better examined if the computation overhead is measured during the real-world flight.

### 5.3. Comparative Analysis with the Prior Studies

We analyze our fault detection model’s key takeaways to be more specifically highlighted when illustrated with previously proposed approaches. Suppose the case when an engineer should establish a fault detection model for the UAV. Among various fault detection studies, the engineer would choose an adequate methodology considering the UAV’s specifications and status quo. We infer that a comparative study of our fault detection approach with previous works can better provide the model’s advantages and disadvantages. Therefore, the engineer can refer to the study to figure out the optimal choice of research to implement a fault detection model. Under the benchmark datasets and accurate ground truths, we shall implement previously-presented fault detection approaches and compare the detection performance.

## 6. Conclusions

In pursuit of a wide range of benefits to society, UAV technology has been highlighted for decades. Although the UAV enables various applications in society, there exists a concern on the safety and reliability. Because UAVs’ fault can cause severe damage, the fault detection system on the UAV is highly required before the UAVs are integrated into society. In response to the aforementioned necessity, prior studies proposed rule-based approaches and supervised learning-based approaches. The suggested approaches achieved a significant fault detection performance; however, rule-based approaches required engineers to update rules whenever finding new types of fault. The supervised learning-based methods necessitated engineers to acquire a finely-labeled dataset to train the detection model, conferring the burden of data acquisition effort. Furthermore, both of the approaches include a common limit that the model can only identify faults trained during the training phase, but fails when novel types of faults are provided to the model.

In this study, we cast the fault detection task as anomaly detection and proposed a fault detection approach leveraging unsupervised learning to resolve prior approaches’ limits. The contributions of our work is provided, as follows. First, we proposed a series of analyses to extract an essential set of features from raw flight logs to identify faulty states’ distinct patterns from safe states. Second, we established a fault detection model that consisted of the stacked autoencoder and trained the model with feature vectors extracted from safe flights. During the inference phase, we expected that the trained model produces a large reconstruction loss on faulty states, and a lower loss on safe states, as the model only understands the pattern of safe states. Finally, we analyzed the difference of reconstruction loss as an effective cue of fault detection and examined our approach’s promising performance in two datasets. While our previous work [19] established a conceptual proposition on fault detection, this study extended prior work by employing an additional dataset and illustrated the detection performance with a proper evaluation metric.

Along with the previously-proposed architecture of real-world fault detection in UAVs [29], we expect that our approach can be easily integrated into the system. Suppose the scenario where a UAV communicates with the base station to receive control commands. We analyzed whether the proposed fault detection approach can recognize UAV’s faulty states by receiving state information from the vehicle or the base station, producing the reconstruction loss from the trained autoencoder, and thresholding the produced loss as compared to the preset threshold. If the UAV navigates autonomously without communications from the base station, we expect that our approah can be embedded to the vehicle and diagnose itself regarding the existence of faults during the flight. By extending our study on detection performance improvement and examining computation overheads, we expect the proposed fault detection model to contribute to acquiring the benefits of UAV in society.

## Figures and Tables

**Table 1:** Features extracted from the UAV Attack (UA) dataset.

Category	Feature Name	Description
Position & Orientation	Local Position (x, y, z)	Local position of the UAV in the local coordinate frame along with the axis x, y, z, respectively
	Ground Speed X	Ground X speed toward the latitude, positive north
	Ground Speed Y	Ground Y speed toward the longitude, positive east
	Ground Speed Z	Ground Z speed toward the altitude, positive down
	Roll	A roll angle
	Pitch	A pitch angle
	Yaw	A yaw angle
	Roll Speed	An angular speed at the roll
	Pitch Speed	An angular speed at the pitch
	Yaw Speed	An angular speed at the speed
	Relative Altitude	An altitude above the home position
	Local Altitude	An altitude in the local coordinate frame
	Quaternion (1, 2, 3, 4)	Quaternion component of w, x, y, z, respectively
IMUs	Acceleration (x, y, z)	An acceleration at axis x, y, z, respectively
	Angular Speed (x, y, z)	An angular speed around axis x, y, z, respectively
	Magnetic Field (x, y, z)	A value of magnetic field at axis x, y, z, respectively
	Absolute Pressure	An absolute pressure at the UAV
	Pressure Altitude	A value of the altitude calculated from the pressure
System Status	Temperature	A temperature of the battery
	Air Speed	Current indicated airspeed
	Heading	Current heading in a compass units scaled in 0 to 360
	Throttle	Current setting of the throttle scaled in 0 to 100
	Climb Rate	Current level of the climb rate

**Table 2:** Features extracted from the ALFA dataset.

Category	Feature Name	Description
Position & Orientation	Velocity (x, y, z)	Measured velocity of axis x, y, z, respectively
IMUs	Angular Velocity (x, y, z)	An angular velocity at axis x, y, z, respectively
	Linear Acceleration (x, y, z)	A linear acceleration at axis x, y, z, respectively
	Magnetic Field (x, y, z)	A value of magnetic field at axis x, y, z, respectively
	Fluid Pressure	A value of the pressure using fluid pressure sensors
System Status	Temperature	A temperature of the battery
	Altitude Error	An error value of current altitude
	Airspeed Error	An error value of current airspeed
	Tracking Error (x)	A tracking error at *x* axis
	WP Distance	A distance between ideal location and current location

**Table 3:** Fault detection result on the flight without any recovery measures. Our approach achieved significant fault detection performances in both fault types.

Fault Type	Number of Safe Logs	Number of Faulty Logs	AUC
GPS Spoofing Attack	2368	41	0.9969
DoS Attack	6203	45	0.9632

**Table 4:** Fault detection results on the flight with recovery measures. Our approach accomplished significant performances in detecting failures on the rudder and elevator, but required further improvements in identifying failures on the aileron and the elevator.

Fault Type	Number of Safe Logs	Number of Fault Logs	AUC
Rudder Failure	207	345	0.9321
Elevator Failure	253	355	0.8076
Aileron Failure	263	545	0.7509
Engine Failure	199	297	0.7646

## Data Availability

UA dataset is available at https://dx.doi.org/10.21227/00dg-0d12 (accessed on 31 January 2021), and ALFA dataset is available at https://doi.org/10.1184/R1/12707963.v1 (accessed on 31 January 2021).
